# Supplementary material for: Targeting Mitochondria by SS-31 Ameliorates the Whole Body Energy Status in Cancer- and Chemotherapy-Induced Cachexia
Source: Cancers (Basel). 2021 Feb 18;13(4):850. doi: 10.3390/cancers13040850 (PMC7923037; doi:10.3390/cancers13040850)
Supplement: Supplementary file 1 [file cancers-13-00850-s001.zip › supplementary/cancers-1100056-supplementary-final.docx]

Supplementary Material: Targeting Mitochondria by SS-31 Ameliorates the Whole Body Energy Status in Cancer- and Chemotherapy-Induced Cachexia

Riccardo Ballarò, Patrizia Lopalco, Valentina Audrito, Marc Beltrà, Fabrizio Pin, Roberto Angelini, Paola Costelli, Angela Corcelli, Andrea Bonetto, Hazel H. Szeto, Thomas M. O’Connell and Fabio Penna


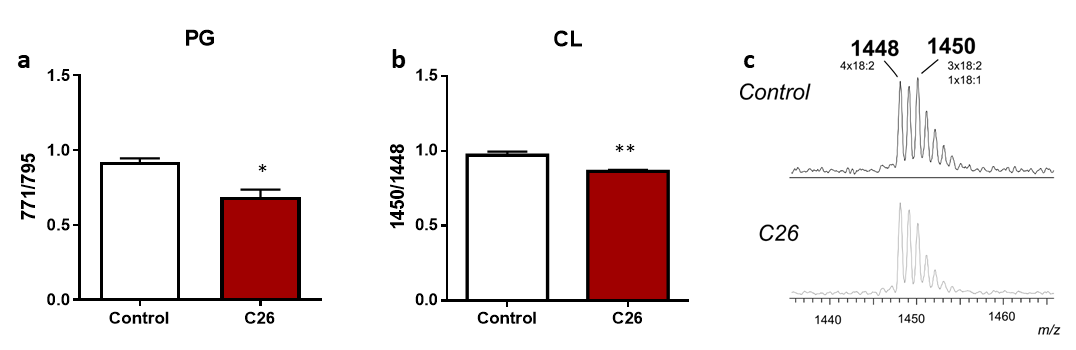


**Figure S1.** Phosphatidylglycerol and cardiolipin mass spectrometry in muscle mitochondria from C26 tumor-bearing mice. MALDI-TOF/MS analysis of phosphatidylglycerol (PG; **a**) and cardiolipin (CL; **b**) content in the gastrocnemius of control (*n* = 7) and C26 mice (*n* = 7). PG levels are expressed as ratio between m/z peak 771 (corresponding to PG 36:3, carrying 18:1 and 18:2 chains) and 795 (corresponding to PG 38:5, carrying 18:1 and 20:4 chains). CL levels are expressed as ratio between m/z peak 1450 (corresponding to CL with three 18:2 chains and one oleic 18:1 chain) and 1448 (corresponding to CL with four linoleic 18:2 chains). Non-parametric Mann-Whitney test used for unpaired comparison for non-normally distributed data. Significance of the differences: * *p* < 0.05, ** *p* < 0.01 vs. control. (**c**) Representative m/z range of CL mass spectra of control (upper mass spectrum) and C26 (lower mass spectrum).


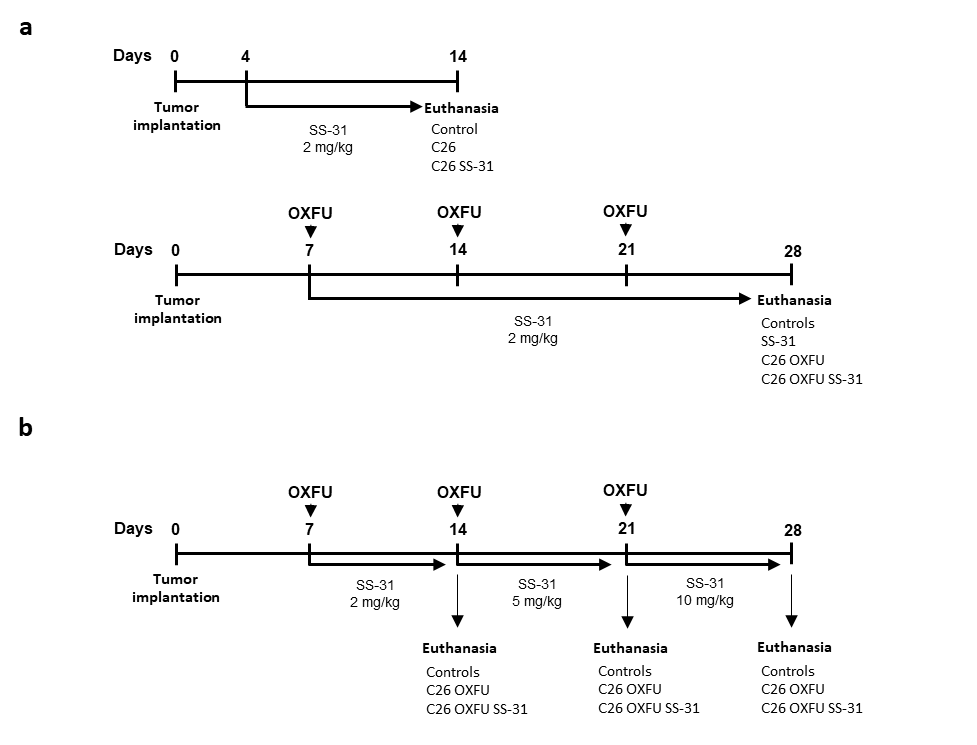


**Figure S2.** Experimental conditions adopted in the current study. Experimental protocol for (**a**) SS-31 administration upon unrestricted tumor growth (C26) or chemotherapy (C26 OXFU) and (**b**) SS-31 increasing dosage in C26 OXFU mice.


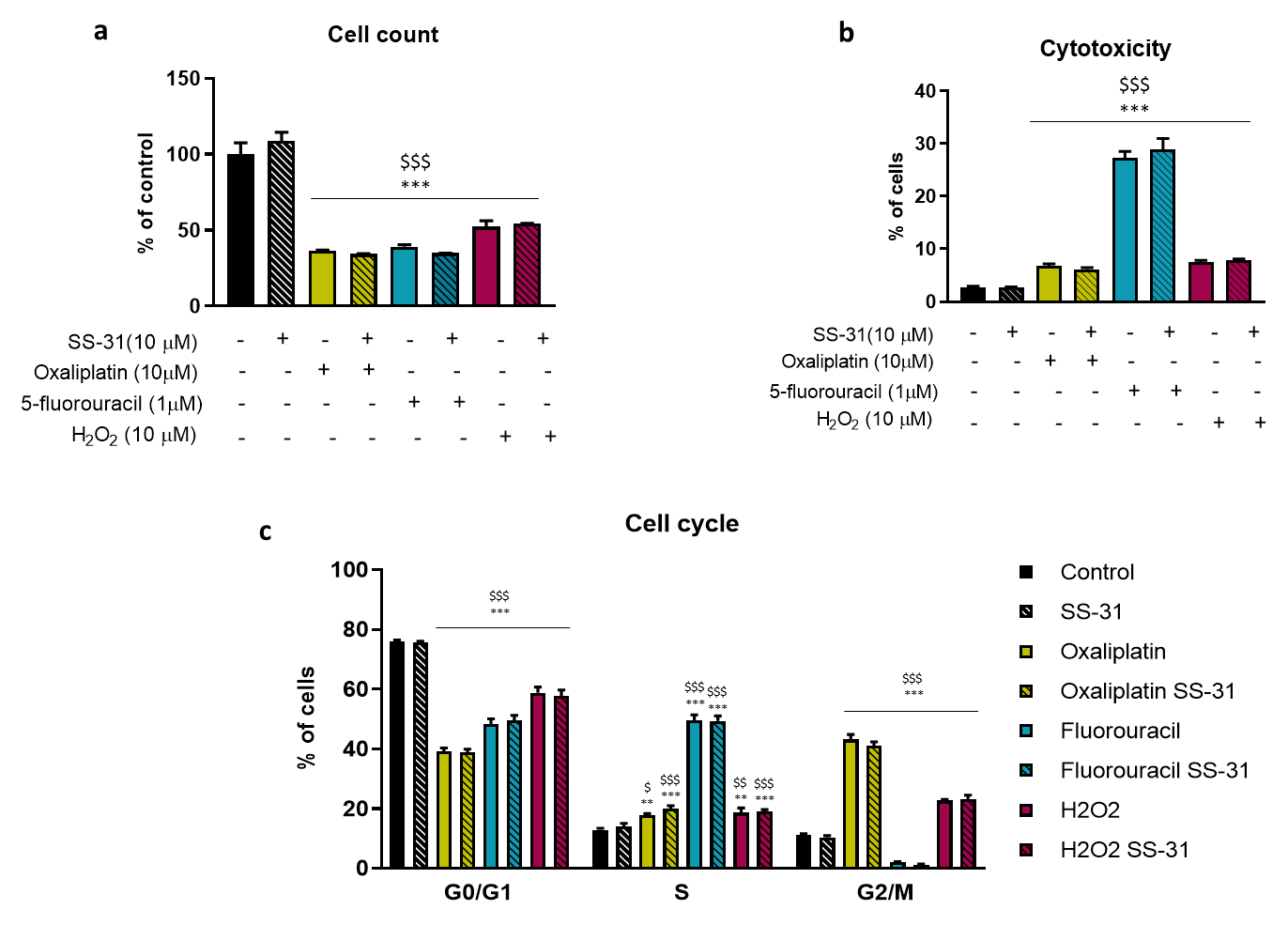


**Figure S3.** SS-31 does not interfere with chemotherapy-induced C26 cell death in vitro. Cell count (**a**), cytotoxicity (**b**) and cell cycle analyses (**c**) of C26 cells treated with oxaliplatin (10 µM), 5-fluorouracil (1 µM) and H_2_O_2_ (10 µM) with or without SS-31 pre-treatment. Cell count data (means ± SD) are expressed as percentage of control (no treatment; black bar). Cytotoxicity and cell cycle data (means ± SD) are expressed as percentage of total cells. The experiment was performed in triplicate for each condition. Significance of the differences: ** *p* < 0.01, *** *p* < 0.001 vs. control; $ *p* < 0.05, $$ *p* < 0.01, $$$ *p* < 0.001 vs. SS-31 alone.


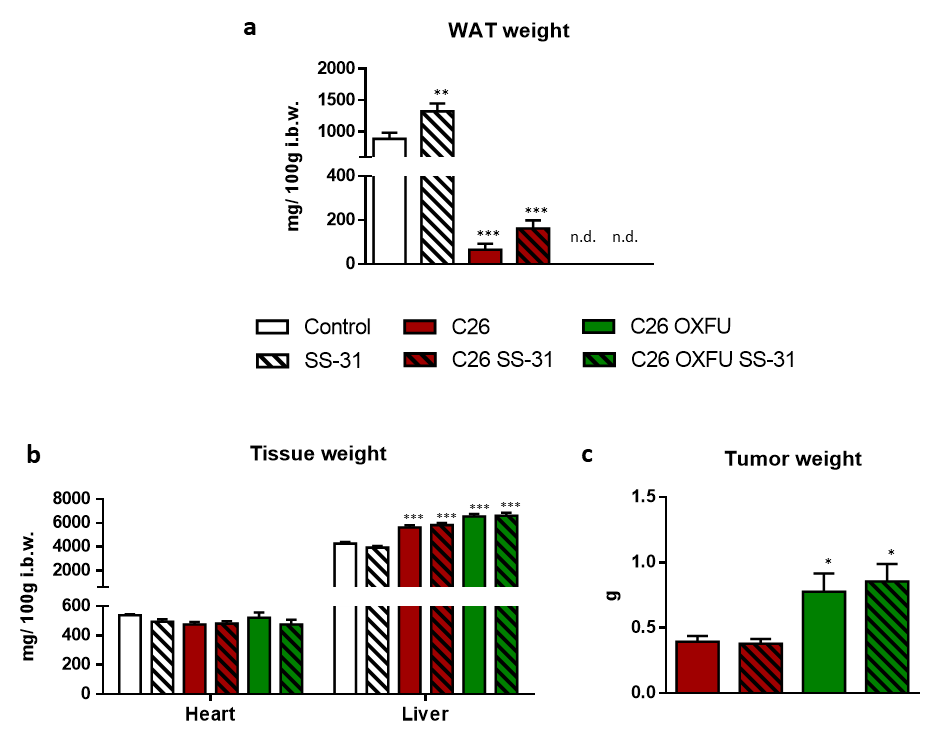


**Figure S4.** Effect of SS-31 on WAT, heart, liver and tumor in C26-bearing mice either receiving chemotherapy or not. White adipose tissue (WAT; **a**), tissue (**b**) and tumor (**c**) weight of controls (*n* = 7), SS-31-treated mice (SS-31; *n* = 6), tumor-bearing mice (C26; *n* = 8), SS-31-treated tumor-bearing mice (C26 SS-31; *n* = 8) and tumor-bearing mice administered with chemotherapy alone (C26 OXFU; *n* = 8) or in combination with SS-31 (C26 OXFU SS-31; *n* = 8). WAT and tissue weight (means ± SEM) are expressed as mg of tissue per 100g of initial body weight. Tumor weight (means ± SEM) is expressed in g. Significance of the differences: * *p* < 0.05,** *p* < 0.01,****p* < 0.001 vs. control; n.d. : not detected.


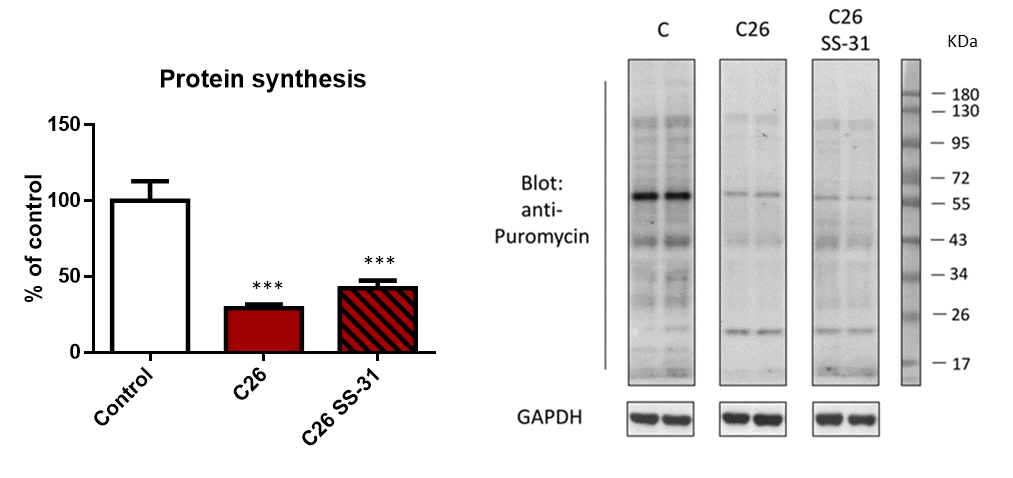


**Figure S5.** SS-31 is unable to restore muscle protein synthesis. Surface sensing of translation (SUnSET) assay (quantification and representative blot) in the skeletal muscle of controls (*n* = 7), tumor-bearing mice (C26; *n* = 8) and SS-31-treated tumor-bearing mice (C26 SS-31; *n* = 8). Data (means ± SEM) are expressed as a percentage of control. Significance of the differences: ****p* < 0.01 vs. control.


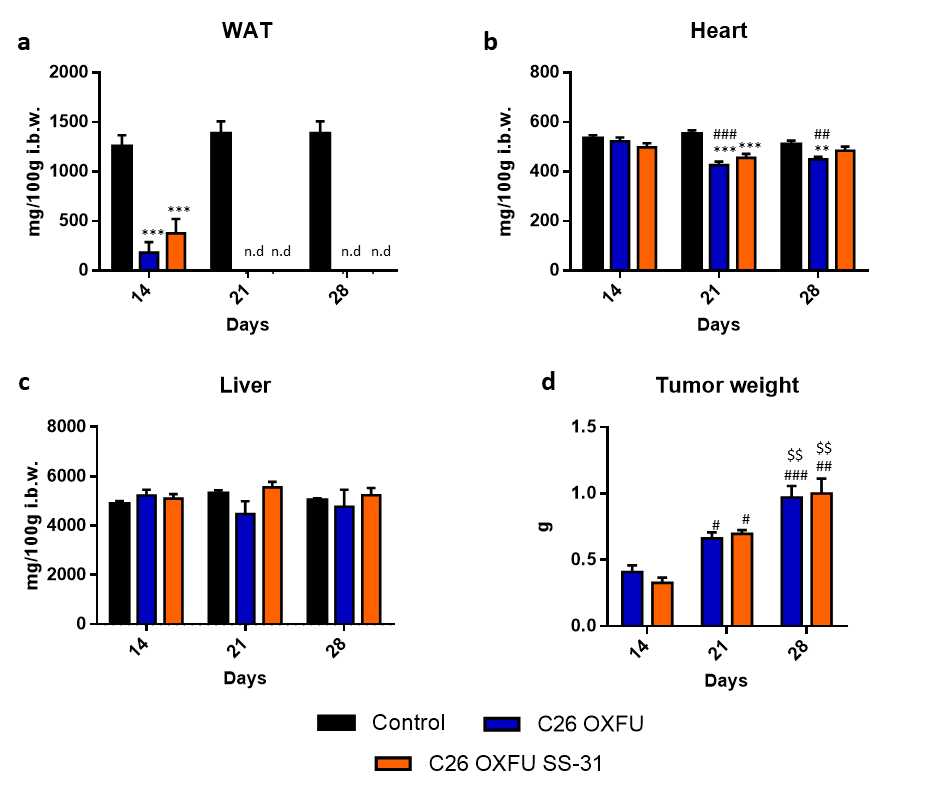


**Figure S6.** Effect of SS-31 on WAT, heart, liver and tumor in C26-bearing mice receiving chemotherapy. White adipose tissue (WAT; **a**), heart (**b**), liver (**c**) and tumor (**d**) weight of controls (*n* = 7) and tumor-bearing mice administered with chemotherapy alone (C26 OXFU; *n* = 8) or in combination with SS-31 (C26 OXFU SS-31; *n* = 8) euthanized 14, 21 or 28 days after tumor implantation. WAT, heart and liver weight (means ± SEM) are expressed as mg of tissue per 100g of initial body weight. Tumor weight (means ± SEM) is expressed in g. Significance of the differences: ***p* < 0.01, *** *p*< 0.001 vs. control; ## *p* < 0.01 vs. day 14; $$ *p* < 0.01 vs. day 21; n.d. : not detected.


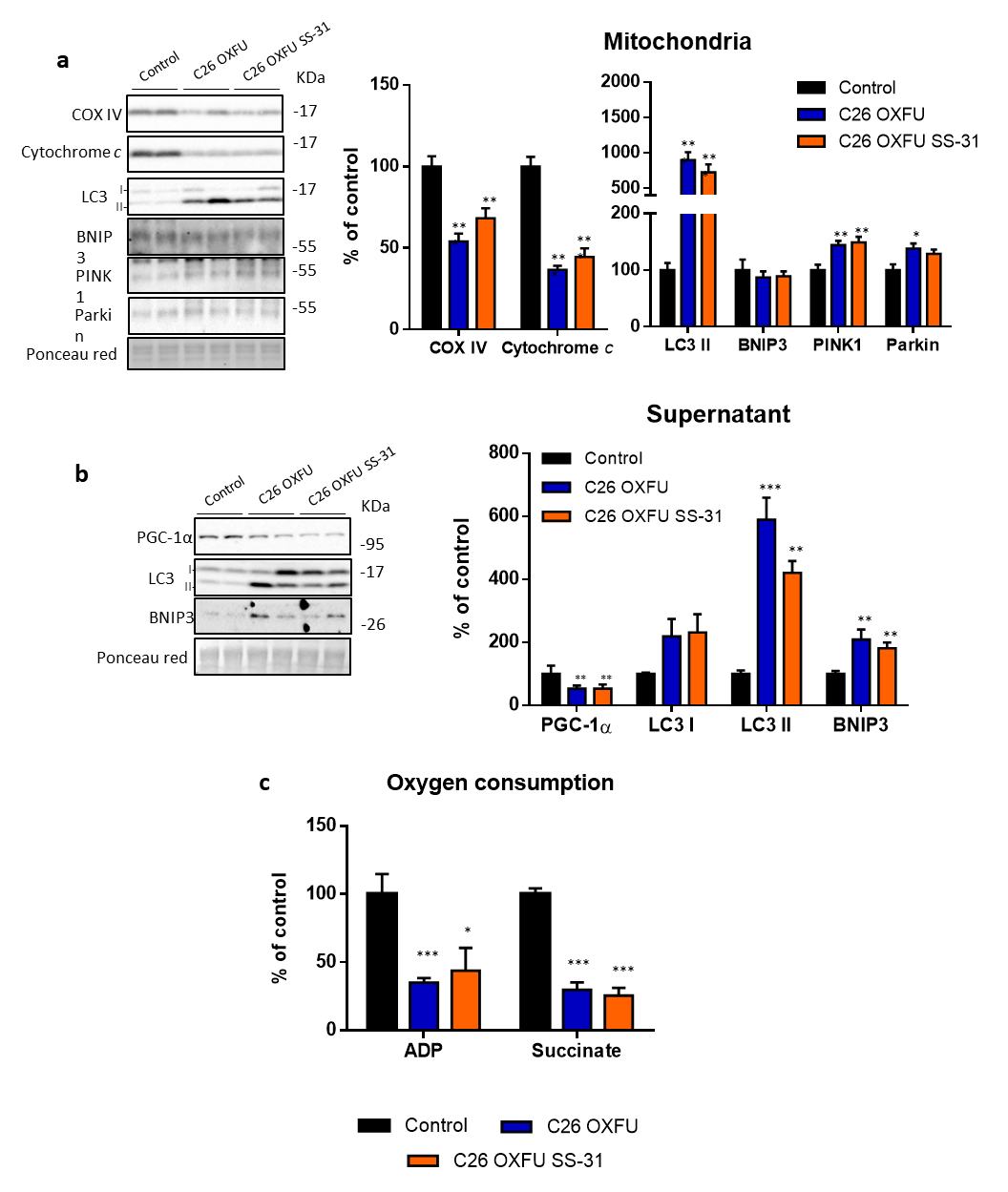


**Figure S7.** SS-31 is no more active on mitochondria and autophagy/mitophagy at 28 day time-point. Mitochondria and autophagy/mitophagy marker proteins (representative blot and quantification) of mitochondrial (**a**) or cytoplasmic (**b**) fraction and ADP/succinate-coupled oxygen consumption (**c**) of controls (*n* = 7) and tumor-bearing mice administered with chemotherapy alone (C26 OXFU; *n* = 8) or in combination with SS-31 (C26 OXFU SS-31; *n* = 8) at day 28 after tumor implantation. Protein and oxygen consumption data (means ± SEM) are expressed as percentage of control. Significance of the difference: * *p*< 0.05, ** *p*< 0.01, *** *p*< 0.001 vs. control.


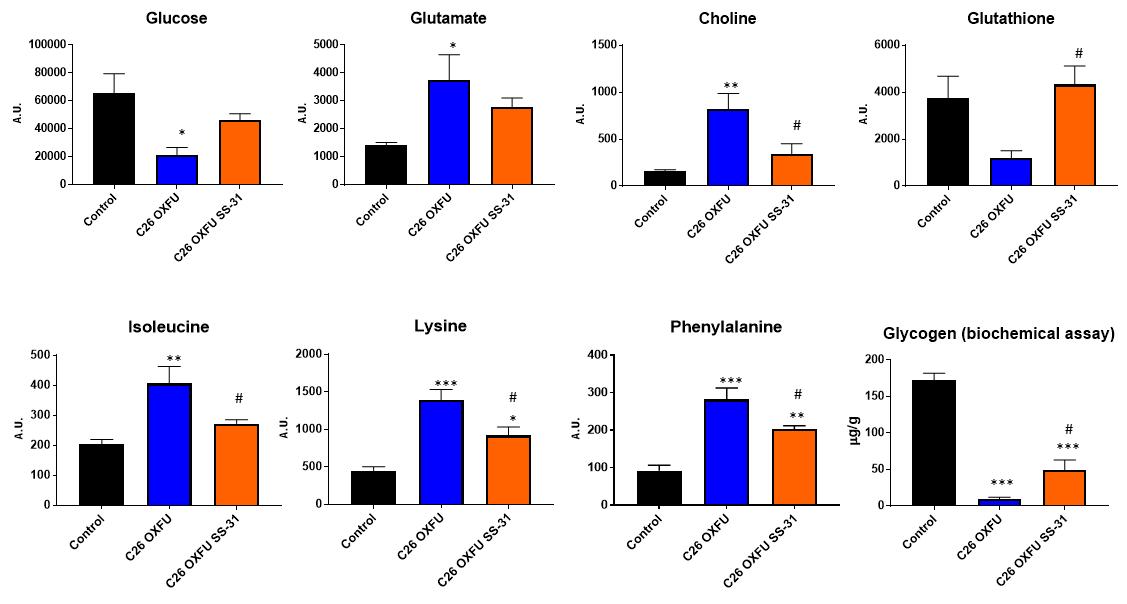


**Figure S8.** SS-31 impacts on liver metabolome of C26-bearing mice receiving OXFU. Liver metabolite levels in controls (*n* = 7) and tumor-bearing mice administered with chemotherapy alone (C26 OXFU; *n* = 8) or in combination with SS-31 (C26 OXFU SS-31; *n* = 8) at day 21 after tumor implantation. Data (means ± SEM) are expressed as arbitrary units (A.U.), except for glycogen content expressed as µg per g of total proteins. Significance of the difference: * *p*< 0.05, ** *p*< 0.01, *** *p*< 0.001 vs. control; # *p* < 0.05 vs.C26 OXFU.


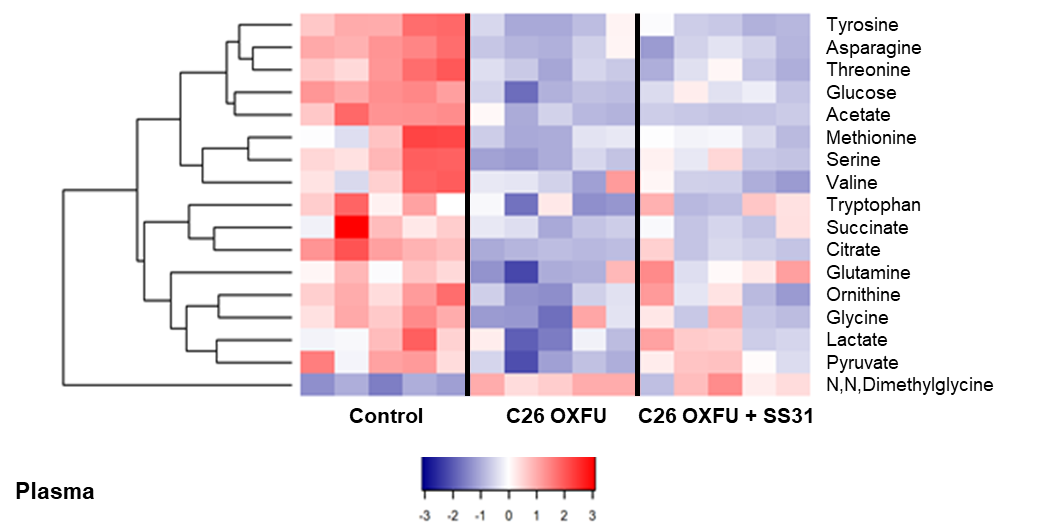


**Figure S9.** Plasma metabolome is moderately affected by SS-31 administration. Heatmap of plasma metabolites of controls (*n* = 5) and tumor-bearing mice administered with chemotherapy alone (C26 OXFU; *n* = 5) or in combination with SS-31 (C26 OXFU + SS-31; *n* = 5) at day 21 after tumor implantation. Colors represent z-scores and metabolites are presented with hierarchical cluster analysis.
